# Supplementary material for: Transcriptome Analysis Reveals Vimentin-Induced Disruption of Cell–Cell Associations Augments Breast Cancer Cell Migration
Source: Cells. 2022 Dec 13;11(24):4035. doi: 10.3390/cells11244035 (PMC9776984; doi:10.3390/cells11244035)
Supplement: Supplementary file 1 [file cells-11-04035-s001.zip › DEGs and enrichment analysis.zip!/DEGs and enrichment analysis/WT_VIMvsVector GO enrichment/GSEA/WT_VIM/INTERMEDIATE_FILAMENT_BASED_PROCESS/INTERMEDIATE_FILAMENT_BASED_PROCESS(GO_0045103).html]

Details for gene set INTERMEDIATE\_FILAMENT\_BASED\_PROCESS(GO:0045103)[GSEA]


|  || Dataset | fpkm.sample |
| Phenotype | sample.cls |
| Upregulated in class | WT\_VIM |
| GeneSet | INTERMEDIATE\_FILAMENT\_BASED\_PROCESS(GO:0045103) |
| Enrichment Score (ES) | 0.93716794 |
| Normalized Enrichment Score (NES) | 1.4648508 |
| Nominal p-value | 0.0 |
| FDR q-value | 0.16299991 |
| FWER p-Value | 0.163 |
Table: GSEA Results Summary

  

Fig 1: Enrichment plot: INTERMEDIATE\_FILAMENT\_BASED\_PROCESS(GO:0045103)
  
Profile of the Running ES Score & Positions of GeneSet Members on the Rank Ordered List

  

| PROBE | DESCRIPTION   (from dataset) | GENE SYMBOL | GENE\_TITLE | RANK IN GENE LIST | RANK METRIC SCORE | RUNNING ES | CORE ENRICHMENT || 1 | ENSG00000186442 | KRT3 |  |  | 1666 | Infinity | -0.0282 | Yes |
| 2 | ENSG00000175084 | DES |  |  | 2208 | Infinity | -0.0373 | Yes |
| 3 | ENSG00000026025 | VIM |  |  | 2388 | 1709.539 | 0.9372 | Yes |
| 4 | ENSG00000132688 | NES |  |  | 2798 | 5.994 | 0.9337 | No |
| 5 | ENSG00000081277 | PKP1 |  |  | 4663 | 2.579 | 0.9036 | No |
| 6 | ENSG00000170819 | BFSP2 |  |  | 6181 | 1.992 | 0.8791 | No |
| 7 | ENSG00000100285 | NEFH |  |  | 6771 | 1.860 | 0.8702 | No |
| 8 | ENSG00000162520 | SYNC |  |  | 7674 | 1.682 | 0.8559 | No |
| 9 | ENSG00000171403 | KRT9 |  |  | 7795 | 1.660 | 0.8548 | No |
| 10 | ENSG00000180921 | FAM83H |  |  | 7944 | 1.632 | 0.8532 | No |
| 11 | ENSG00000136827 | TOR1A |  |  | 8182 | 1.597 | 0.8501 | No |
| 12 | ENSG00000142168 | SOD1 |  |  | 8206 | 1.593 | 0.8506 | No |
| 13 | ENSG00000164690 | SHH |  |  | 8548 | 1.541 | 0.8457 | No |
| 14 | ENSG00000111057 | KRT18 |  |  | 10205 | 1.334 | 0.8185 | No |
| 15 | ENSG00000186847 | KRT14 |  |  | 11204 | 1.247 | 0.8023 | No |
| 16 | ENSG00000057294 | PKP2 |  |  | 12177 | 1.167 | 0.7865 | No |
| 17 | ENSG00000173744 | AGFG1 |  |  | 12722 | 1.123 | 0.7780 | No |
| 18 | ENSG00000182253 | SYNM |  |  | 13149 | 1.091 | 0.7714 | No |
| 19 | ENSG00000132155 | RAF1 |  |  | 13338 | 1.077 | 0.7688 | No |
| 20 | ENSG00000131095 | GFAP |  |  | 14245 | 1.007 | 0.7541 | No |
| 21 | ENSG00000170465 | KRT6C |  |  | 14562 | 0.996 | 0.7493 | No |
| 22 | ENSG00000186832 | KRT16 |  |  | 15363 | 0.949 | 0.7363 | No |
| 23 | ENSG00000066427 | ATXN3 |  |  | 18354 | 0.779 | 0.6861 | No |
| 24 | ENSG00000096696 | DSP |  |  | 19375 | 0.721 | 0.6693 | No |
| 25 | ENSG00000166579 | NDEL1 |  |  | 19648 | 0.708 | 0.6651 | No |
| 26 | ENSG00000113712 | CSNK1A1 |  |  | 19674 | 0.706 | 0.6651 | No |
| 27 | ENSG00000132932 | ATP8A2 |  |  | 19929 | 0.693 | 0.6612 | No |
| 28 | ENSG00000171100 | MTM1 |  |  | 20482 | 0.662 | 0.6522 | No |
| 29 | ENSG00000105993 | DNAJB6 |  |  | 20888 | 0.641 | 0.6457 | No |
| 30 | ENSG00000112851 | ERBIN |  |  | 21585 | 0.603 | 0.6343 | No |
| 31 | ENSG00000151914 | DST |  |  | 23103 | 0.498 | 0.6089 | No |
| 32 | ENSG00000143952 | VPS54 |  |  | 23943 | 0.442 | 0.5950 | No |
| 33 | ENSG00000128422 | KRT17 |  |  | 25113 | 0.336 | 0.5754 | No |
| 34 | ENSG00000114796 | KLHL24 |  |  | 25534 | 0.305 | 0.5684 | No |
| 35 | ENSG00000170484 | KRT74 |  |  | 27669 | 0.000 | 0.5323 | No |
| 36 | ENSG00000172867 | KRT2 |  |  | 29036 | 0.000 | 0.5092 | No |
| 37 | ENSG00000139648 | KRT71 |  |  | 33878 | NaN | 0.4273 | No |
| 38 | ENSG00000148798 | INA |  |  | 37591 | NaN | 0.3645 | No |
| 39 | ENSG00000104722 | NEFM |  |  | 50333 | NaN | 0.1489 | No |
| 40 | ENSG00000204897 | KRT25 |  |  | 55079 | NaN | 0.0686 | No |
| 41 | ENSG00000277586 | NEFL |  |  | 56226 | NaN | 0.0492 | No |
| 42 | ENSG00000171431 | KRT20 |  |  | 59046 | NaN | 0.0015 | No |
Table: GSEA details
[plain text format]

  

Fig 2: INTERMEDIATE\_FILAMENT\_BASED\_PROCESS(GO:0045103)
  
Blue-Pink O' Gram in the Space of the Analyzed GeneSet

  

Fig 3: INTERMEDIATE\_FILAMENT\_BASED\_PROCESS(GO:0045103): Random ES distribution
  
Gene set null distribution of ES for
 **INTERMEDIATE\_FILAMENT\_BASED\_PROCESS(GO:0045103)**
